# Peri-implant soft tissue conditioning of immediate posterior implants by CAD-CAM socket sealing abutments: a randomized clinical trial

**DOI:** 10.1186/s12903-024-05417-w

**Published:** 2025-01-17

**Authors:** Mai Mohamed Elgendi, Iman Salah Eldin Hamdy, Hanaa Ibraheem Sallam

**Affiliations:** 1 Department of Fixed Prosthodontics, Faculty of Dentistry, Cairo University, Cairo, Egypt; 2 Division of Fixed Prosthodontics, Department of Prosthetic Dentistry, Faculty of Dentistry, King Salman International University, Al Tur, Egypt

**Keywords:** Immediate posterior implants, Socket sealing abutments, Standard healing abutments, Peri-implant soft tissue profile, CAD-CAM, PEEK

## Abstract

**Background:**

Anatomically formed healing abutments were suggested in literature to address many of the issues associated with immediate posterior implant insertion such as large extraction sockets that are extremely hard to seal without reflecting the mucoperiosteal flap, extraction sockets anatomy that are not suitable for regular healing abutment placement, and potentially high occlusal stresses when planning a temporary implant supported prothesis to improve the conditioning of supra implant tissue architecture and the emergence profile of the implant supported restorations.

**Purpose:**

To clinically evaluate the peri-implant soft tissue profile of single posterior implant retained restorations and to assess patient related outcomes of the implant restorations that were conditioned immediately by CAD-CAM socket sealing abutments (SSA) versus those conditioned by Titanium (Ti) standard healing abutments (SHA).

**Methods:**

Twenty participants received twenty-two single maxillary immediate implants after flapless minimally invasive tooth extraction and 3D guided implant placement in the posterior area (premolar and molar) and allocated randomly into two groups (*n* = 11), the intervention group: patients received PEEK SSA and the control group: the patients received Ti SHA. Modified Pink Esthetic Score (PES) was evaluated at 3 observation periods: Baseline T0 (immediate after implant supported crown insertion), 6 months T1 and 1 year of clinical performance T2. Patient satisfaction was assessed one week and one year after crown insertion using visual analogue scale (VAS).

**Results:**

At base line, after six as well as 12 months, SSA group showed statistically significant higher total modified PES scores than SHA group (*P*-value < 0.001). At the 2 clinical observation periods (baseline and after one year), SSA group showed statistically significantly higher overall satisfaction score than SHA group (*P*-value < 0.001).

**Conclusion:**

After one year of clinical observation period, CAD-CAM PEEK socket sealing abutments together with flapless minimally invasive tooth extraction and 3D guided implant placement provided superior outcomes compared to Ti SHA in terms of peri-implant soft tissue profile.

**Trial registration:**

This study was registered on clinicaltrials.gov with ID no. NCT05276765 on 03/03/2022.

## Introduction

One of the frequently used treatment approaches in implant dentistry is immediately replacing a severely damaged posterior tooth with a dental implant [1]. This method offers substantial therapeutic advantages for both the patient and the dentist, such as decreased treatment duration and a decrease in frequency and invasiveness of surgical procedures. This technique of tooth replacement has shown favorable results and a high percentage of long-term success [2, 3].

An important obstacle that a clinician might experience while doing immediate insertion in the posterior teeth area, is the difficulty of achieving a completely tight primary closure of the soft tissue. The use of this technical approach is often accompanied by several invasive surgical procedures and substantial postoperative healing such as displacement of the muco-gingival line, periosteal releasing incision flaps, sutures, and membrane stabilization [4].

In order to minimize the invasiveness and sensitivity of the procedure, clinicians used provisional restoration technique to shape the soft tissues into a transmucosal contour that closely resembles natural teeth [5, 6]. However, this approach negatively impacts the primary stability of the implant by exposing it to the potential danger of being loaded prematurely [7].

Conventionally, standard healing abutments were used to condition the peri-implant soft tissues in the second surgical phase after passing the healing period. However, this approach is accompanied by surgical invasiveness such as the need for mucoperiosteal incisions and/ or flap elevation, suturing, displacement of the mucogingival line, and probability of decrease or loss of the keratinized tissues [8].

Recently, the utilization of anatomical healing abutments was suggested to offer benefits in terms of conserving the current soft tissue structure, maintaining the height of the marginal bone, and minimizing the likelihood of early loading of the immediate implant throughout the healing process [9, 10]. The customized healing abutment approach involves the external application of composite resin on a temporary abutment to provide support to the soft tissue and replicate the shape of the final restoration. The drawback of this procedure is the increased amount of time required to create the customized anatomical healing abutment and the risk of contamination of the socket with excess resin composite material throughout the customization process [11].

In modern dentistry, the use of digital technology has led to the proposal of a technique involving the creation of a CAD-CAM healing abutment either before or immediately after the dental implant surgery. This abutment is designed to facilitate the healing process by following the shape of the extraction socket, providing support to the soft tissues to prevent their collapse during healing, stabilizing the blood clot, creating space for bone regeneration, and developing a prosthetic emergence profile that mimics the natural tooth's anatomy [10–12].

However, the literature lacks sufficient data assessing the clinical advantages of using CAD-CAM socket sealing abutments in preserving the peri-implant soft tissue profile and studying the direct impact of using them on the patient related outcomes and their overall satisfaction. Therefore, the aim of this study is to assess and compare the soft tissue profile and patient related outcomes of immediate implant restorations placed in the maxillary posterior area that were customized using CAD-CAM socket sealing abutments versus those customized using Ti standard healing abutments.

The study null hypothesis was that there would be no clinical differences in the peri-implant soft tissue profile and patient related outcomes of implant supported restorations conditioned by means of CAD-CAM socket sealing abutments and those conditioned by Ti standard healing abutments.

## Material and methods

### Study design

The current study was a monocentric randomized controlled clinical trial (RCT) with parallel group, in which twenty recruited participants were received twenty-two single maxillary immediate implants in the posterior area (premolar and molar) and allocated randomly into two groups according to the type of healing abutment: the intervention group: patients received CAD-CAM PEEK socket sealing abutments (SSA) and the control group: the patients received Ti standard healing abutments (SHA), taken into consideration that there were 2 patients received 2 implants with the same type of healing abutment in each group. The patients were recruited at the Prosthodontics department, Faculty of Dentistry, Cairo University. The treatment plan was explained to each patient. Then, they agreed to sign the informed consent before proceeding to the clinical work. All the clinical procedures were carried out by one experienced clinician.

The participants were allocated into two groups with 1:1 allocation ratio by using computerized sequence generation (www.randomizer.org) according to type of healing abutment. After the implant was placed and the grafting biomaterial was packed, the opaque well sealed envelope related to the patient's inclusion was opened. The clinician then received information on whether the patients should be assigned to the intervention or the control group.

By adopting an alpha level of (0.05), a beta of (0.2) i.e. power = 80% and a minimal clinically important difference = 1. The minimum estimated sample size was 9 subjects (implants) per group calculated based on the results of Perez et al., Sample size was increased by (20%) to compensate for possible dropouts to be (11) subjects per group [13]. Sample size calculation was approved by the Medical Biostatistics unit at Faculty of Dentistry, Cairo university with approval date.: 11/12/2021. This study and the informed consent template were approved by the Ethics Committee of Scientific Research – Faculty of dentistry- Cairo University- in 25- 1- 2022 with approval no.: 11/1/22. This study was double blinded (assessor, statistician).

Patients selected were characterized as follows:

- Inclusion criteria:
  1. The patient’s age: 18–50 years old.
  2. Non-restorable maxillary posterior teeth with intact adjacent teeth.
  3. Sufficient bone to insert a dental implant with a minimum length of 8 mm and average diameters in premolar and molar areas (3.5- 4 mm) and (5–6 mm), respectively.
  4. Sufficient apical bone (3-5mm) to anchor the implant with minimum primary stability of 30N/cm.
  5. Socket types 1 and 2 according to Steigmann et al., (Intact buccal bone or minimal buccal bone fenestration apically) with minimum 1 mm buccal bone thickness [14].
  6. Minimum 3 mm of attached keratinized buccal mucosa.
  7. Sufficient prosthetic space (mesio‐distal, bucco‐lingual, and 8–12 mm inter-occlusal space) for placement of the definite restoration.
- Exclusion criteria:
  1. Patients who are alcoholic or drug abusers.
  2. Smokers.
  3. Patients with acute periapical infections.
  4. Patients had or undergoing radiotherapy to the head and neck region due to malignancy.
  5. Bruxer patients or having TMJ dysfunction.
  6. Intake of drugs affecting bone metabolism.
  7. Uncontrolled diabetic patients.
  8. Pregnant women.

## Surgical protocol

### Virtual 3D implant planning and construction of 3D printed surgical template

**Fig. 1:**
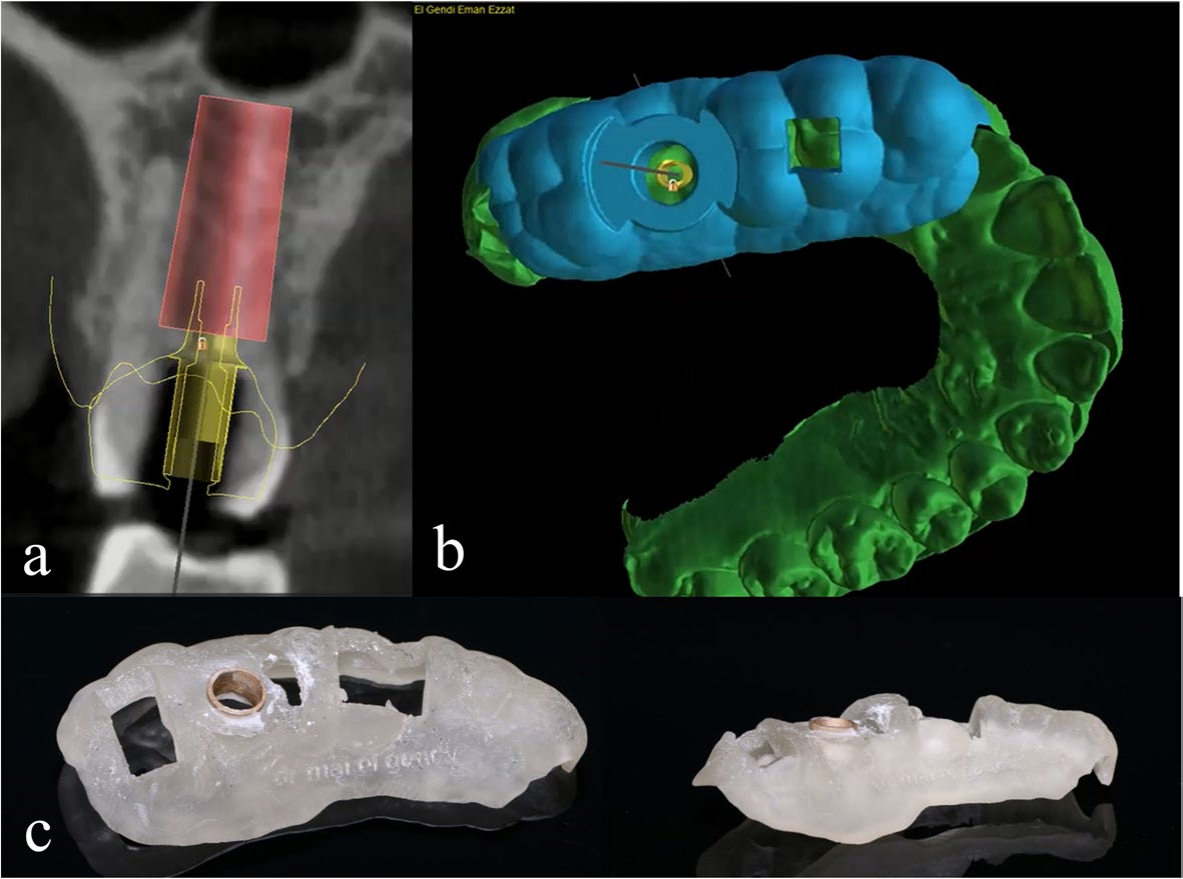
Virtual implant positioning and 3D printed surgical template: **a** Cross-sectional view of a virtual implant at maxillary right first molar area (implant diameter 5.5mm and length 12mm) according to bony architecture and final proposed screw retained crown, **b** design of the surgical template and **c** 3D printed resin surgical template

After clinical and radiographic examination, the Standard Tessellation Language (STL) data obtained from the extraoral scanning of the primary casts by desktop scanner (DOF Swing HD, Nevada) and the Digital Imaging and Communication in Medicine (DICOM) files obtained from the CBCT imaging acquisition unit (Promax 3D, Planmeca, Finland) were imported to the computer-guided planning software (Blue Sky Bio, US) and merged. The optimal site of the implant was virtually planned for immediate insertion according to the bony architecture and the final proposed screw retained crown. A fully guided tooth-supported surgical template was designed based on prosthetically driven implant placement approach and subjected for 3D printing using a stereolithography rapid prototyping machine (AccuFab-D1s, Shining 3D Dental, China) in clear transparent resin Fig. 1.

### Designing and milling of the customized CAD-CAM PEEK socket sealing abutment

**Fig. 2:**
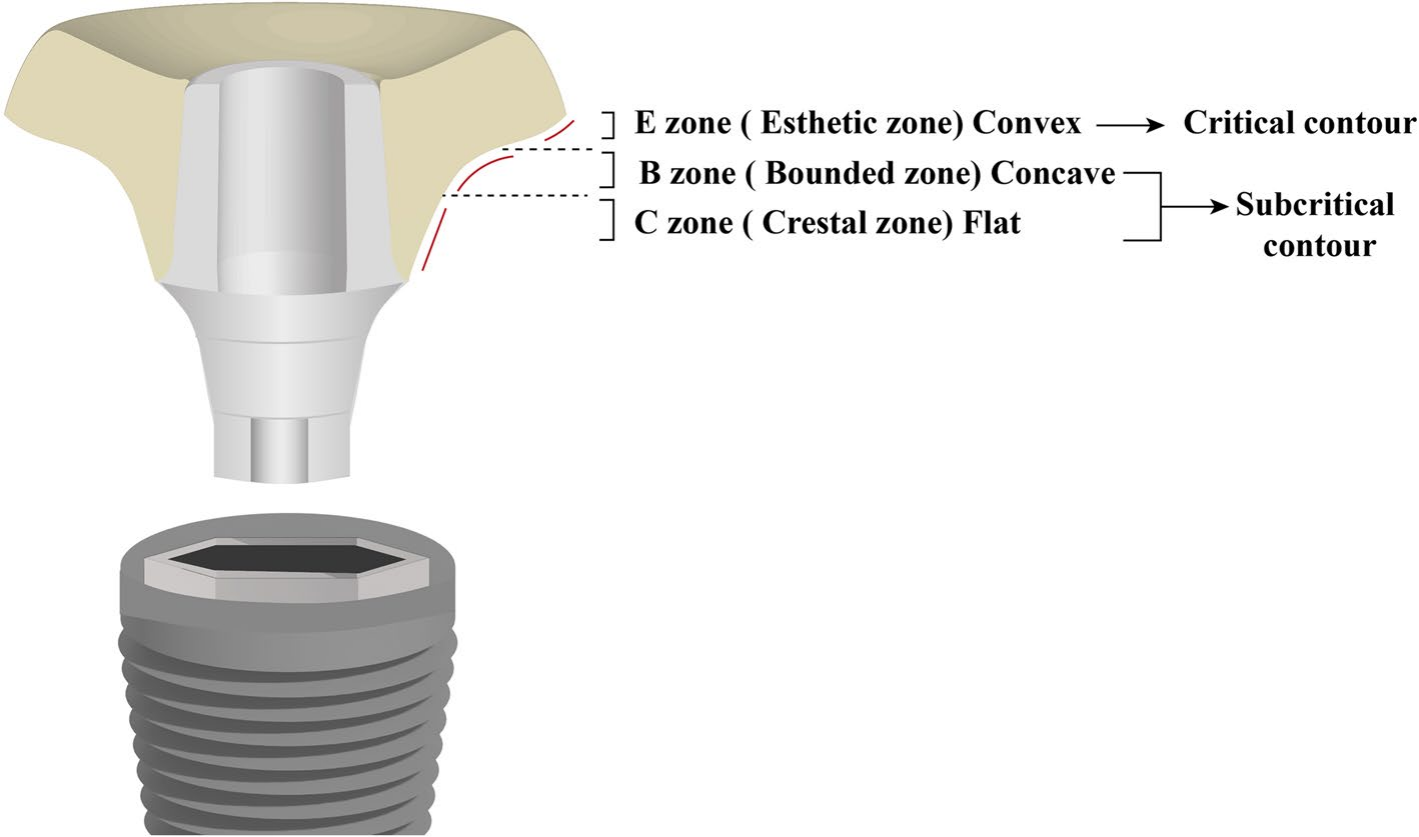
Design of CAD-CAM socket sealing abutment based on EBC concept

This study applied an indirect digital workflow in construction of the PEEK SSA which was prepared for use on the day of surgery. Following the virtual removal of the non-restorable maxillary posterior tooth, the combined STL and DICOM data were imported into CAD software (Blender for Dental (B4D), Australia) then, a full anatomical contoured screw retained implant-supported crown was designed and modified by cutting into the cervical contour to shape the customized socket sealing abutment. The SSA was designed based on the esthetic biological contour (EBC) concept to mimic the contour of the original tooth at the emergence profile level resulting in a socket sealing abutment with a flat, concave, convex contour of 4 mm height corresponds to the soft tissue thickness at the transition zone [15, 16] Fig. 2.

Once the digital design was completed, the socket sealing abutment's core was generated using milling machining (Roland DWX-52D, Korea) of poly-ether-ether-ketone (PEEK) material. PEEK may be used to manufacture implant healing abutments due to its acceptable biocompatibility with human fibroblast cells. Moreover, the adherence of oral microbial flora to PEEK abutments is similar to that observed with titanium, zirconia, and PMMA abutments showing optimum peri-implant soft tissue healing after sufficient healing periods. The similarity in modulus of elasticity between the bone and surface of PEEK minimizes the negative effects of the stresses and promotes remodeling of bone [17]. Therefore, many authors found PEEK as a feasible substitute for titanium in the fabrication of implant healing abutments [9, 18].

The inner surface of the PEEK SSA was sandblasted with 110 μm aluminum oxide at a distance of 10 mm for 10 s and at 2.5 bar pressure using air abrasion machine [19].

Adhesive primer (Visio.link, Bredent, UK) was applied to the inner surface of PEEK after sandblasting and light cured for 90 s [19]. It was then securely bonded to the non-engaging sandblasted prefabricated Ti base abutment (PCOR Ti base. TRATE AG, Switzerland) using dual cured self-adhesive resin cement (PENTRON BREEZE, USA) after sealing the screw access channel with PTFE tape and flowable light cured resin composite.

### Surgical procedures

Patients were administered 4% Articaine local anesthesia (ARTINISBA 4%, Spain) and the tooth extraction procedure was carried out using a flapless and minimally invasive approach by sectioning the existing roots and cutting the supracrestal gingival fibers with periotomes. Subsequently, curettage was performed to remove any infected tissue using a curette, followed by irrigation with physiologic saline and 0.2% chlorohexidine.

**Fig. 3:**
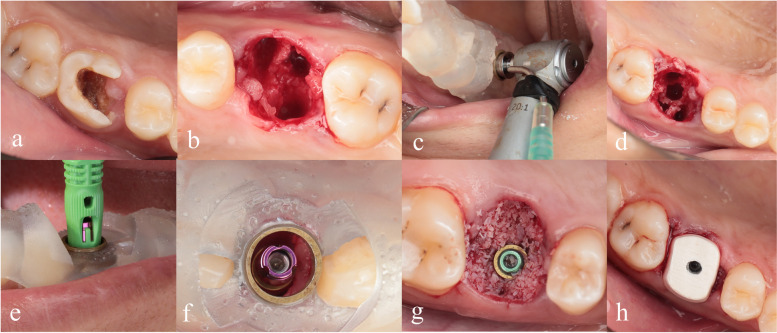
Surgical procedures: **a** Non-restorable maxillary right first molar, **b** clear irrigated socket walls after flapless minimally invasive tooth extraction, **c** guided drilling through surgical template, **d** prepared osteotomy in the interseptal bone, **e** implant insertion through the guide, **f** implant placed in situ, **g** filling the jumping gap with xenograft bone substitute and **h** PEEK SSA acting as a barrier for the xenograft bone material at immediate implants site

After osteotomy preparation in the interseptal bone through the surgical template, platform switched ROOTT implants with morse taper connection were inserted where the distance between the implant platform and the cementoenamel junction of the neighboring teeth was 3mm ensuring the threshold of initial stability was 35N/cm. A cover screw was inserted, and the space between the implant surface and the socket walls was filled with a bone xenograft (Bioteck Bio-Gen Granules, Belgium) to promote healing. Either PEEK SSA or Ti SHA were screwed to the implants according to the allocated group after removal of the cover screw immediately after implant insertion. In SHA group, collagen sponge was packed into the sutured socket and stabilized by the standard healing abutment Fig. 3.

Patients were asked to adhere to a medical regimen for a duration of 3–5 days involving 300mg clindamycin every 12 h, and anti-edematous drug every 12 h.

## Prosthetic protocol

**Fig. 4:**
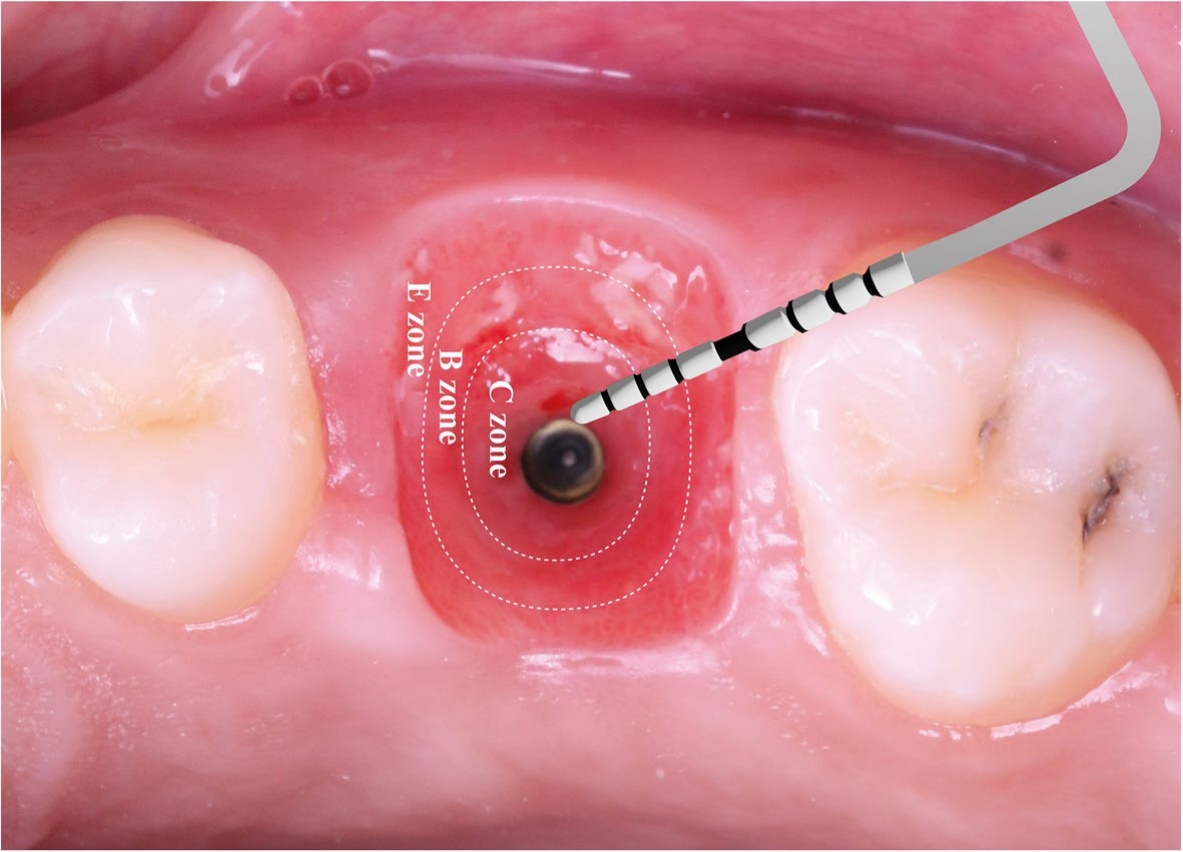
EBC zones represented in the customized peri-implant soft tissue by the CAD-CAM SSA

After passing 6 months of healing, the PEEK SSA was removed to check for the maturation of the peri-implant soft tissue Fig. 4.

**Fig. 5:**
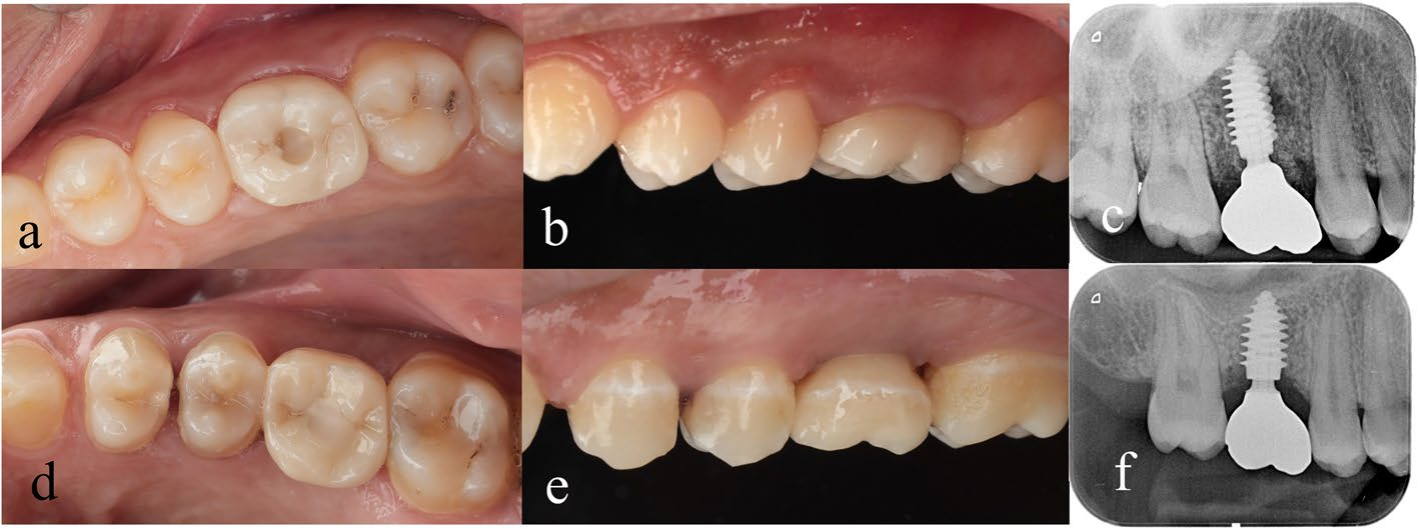
Postoperative baseline photographs showing screw retained zirconia implant crown restoring non-restorable maxillary right first molars: **a** Occlusal view for SSA group, **b** buccal view in black background for SSA group, **c** postoperative periapical radiograph for SSA group, **d** occlusal view for SHA group, **e** buccal view in black background for SHA group and **f **postoperative periapical radiograph for SHA group

The customized open tray impression coping was screwed to the implant after customization by indirect soft tissue impression technique. Then, a single step double consistency (putty and light) addition silicon (polyvinyl siloxane) impression (Zhermack Elite HD + Putty Soft Normal Set, Italy) was employed in both groups. Exocad software program was used to design the screw retained crowns according to EBC concept which were milled in katana super translucent multilayered zirconia (KATANA™ Zirconia STML, Japan). The crown framework was divided into two distinct areas: (1) the subgingival peri-implant soft tissue side area, which was made of ultra polished zirconia to avoid any irritation to the surrounding tissues, and (2) the glazed ceramic area, which begin above the gum line at the point where the restoration emerges from the peri-implant tissues. The fitting surface of the zirconia crown was sandblasted (aluminum oxide Al_2_O_3_, particle size 50 μm, at pressure 2 bar at a 5mm step over distance for 15 s), then cemented to the prefabricated sandblasted engaging Ti base abutment (PCO1 Ti base abutment, TRATE AG, Switzerland) using dual cured self-adhesive resin cement and light cured for 60 s. The screw retained crowns were screwed to the implants at torque of 35 N/cm according to the manufacturer instructions. The screw access channels were filled by sterile PTFE tape and light cured resin composite. Baseline digital photographs and periapical radiographs with parallel technique were taken for all patients in both groups Fig. 5.

## Study outcomes

Postoperative digital photographs were taken after placement of the screw retained implant supported crowns of both groups (baseline T0) and during follow-up sessions (T1 6th months follow up and T2 one year follow up).

### Assessment of peri-implant soft tissue profile

The peri-implant soft tissue profile of twenty-two screw retained implant supported monolithic zirconia crowns in twenty patients was evaluated using the modified Pink Esthetic Score (PES). The obtained data were recorded as a numbering score (0, or 1 or 2) with the total score out of 10.

### Patient related outcomes

The assessment of patient satisfaction was carried out through the use of a questionnaire that was conducted using the visual analogue scale (VAS) 2 times during the research period: baseline T0 (one week after crown insertion) and T1 after one year of clinical observation period. The questionnaire was thoroughly presented to each patient including a detailed discussion of the scoring methodology where zero represented completely dissatisfied and 10 represented completely satisfied. The satisfaction scale assessed the functional parameters such as chewing and cleansing efficiency and esthetic parameters such as shape, color and gum appearance of the implant supported restorations.

### Statistical analysis

Scores data are non-parametric data. Data were presented as median, range, mean and standard deviation (SD) values. Mann–Whitney U test was used to compare between the two groups. Friedman’s test was used to study the changes within each group. Dunn’s test was used for pair-wise comparisons when Friedman’s test is significant. Wilcoxon signed-rank test was used to study the changes in satisfaction scores after 12 months. Qualitative data were presented as frequencies and percentages. The significance level was set at P ≤ 0.05. Statistical analysis was performed with IBM SPSS Statistics for Windows, Version 23.0. Armonk, NY: IBM Corp.

## Results

**Fig. 6:**
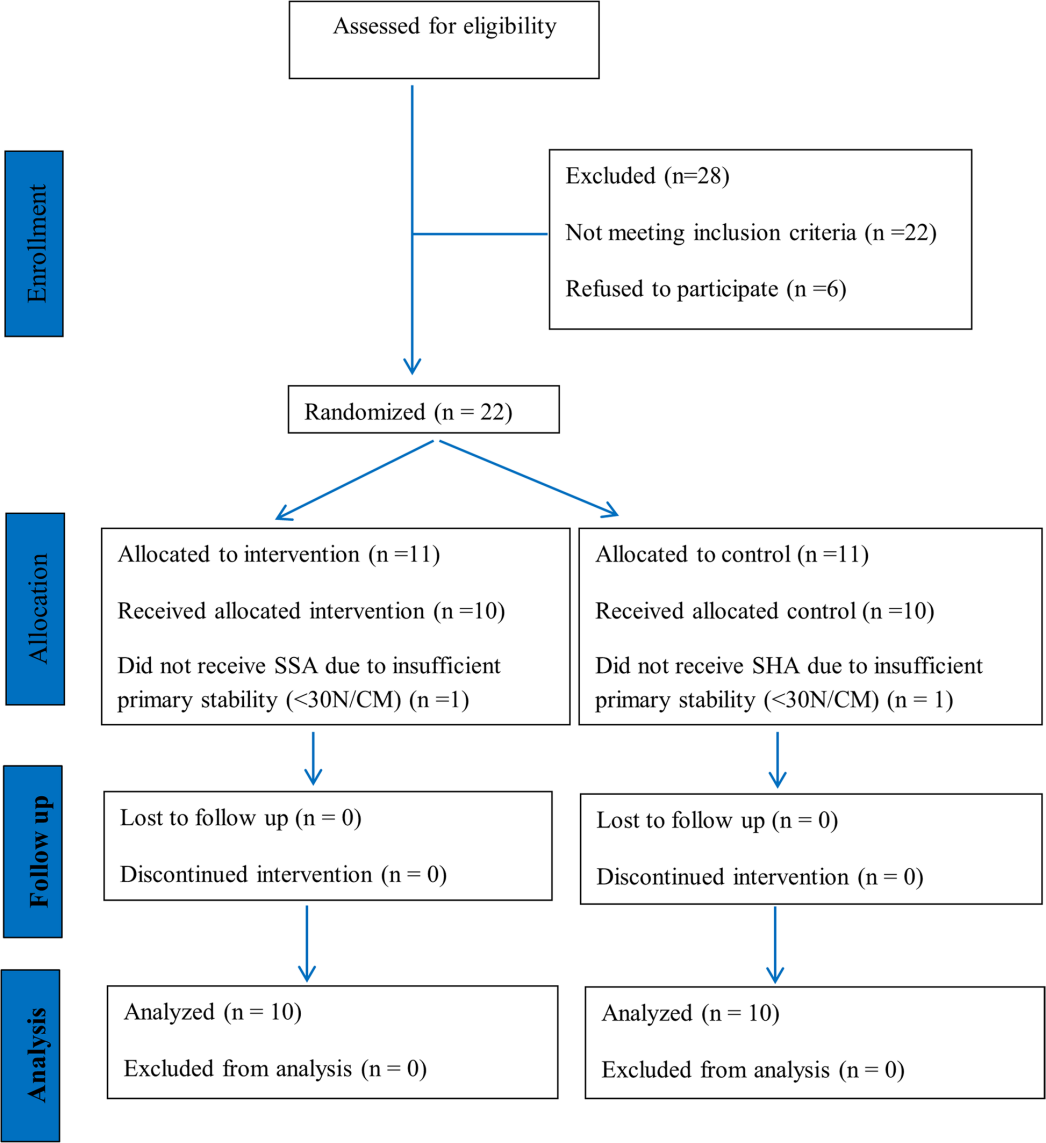
CONSORT flow chart

The patient distribution through each stage of the randomized clinical trial according to the CONSORT statement flow chart is reported in Fig. 6 where n represents number of patients.

## Patients’ demographic data

The patients’ base line characteristics are represented in Table 1.

**Table 1:** Frequencies (n), percentages (%), mean and standard deviation (SD) values for patients’ demographics in the two groups

Base line characteristics	SSA	SHA
**Gender [n, (%)]**		
**Male**	2 (20%)	2 (20%)
**Female**	8 (80%)	8 (80%)
**Age in years [Mean, SD]**	29.6 (8.2)	27.3 (6.7)
**Insertion torque in N/cm [Mean, SD]**	48.5 (5.3)	44.3 (7.3)
**Site [n, (%)]**		
**Premolar**	5 (45.5%)	5 (45.5%)
**Molar**	6 (54.5%)	6 (54.5%)
**Socket type [n, (%)]**		
**ST1**	7 (63.6%)	7 (63.6%)
**ST2**	4 (36.4%)	4 (36.4%)

## Peri-implant soft tissue profile

### Mesial and distal papillae

At base line, after six as well as 12 months, SSA group showed statistically significant higher mesial papilla scores than SHA group (*P*-value = 0.001, Effect size = 1.653), (*P*-value < 0.001, Effect size = 1.922) and (*P*-value < 0.001, Effect size = 1.922) for each observation period, respectively and higher distal papilla scores than SHA group (*P*-value = 0.001, Effect size = 1.564) for all the observation periods.

In both groups, there was no statistically significant change in mesial papilla scores through different time periods (*P*-value = 0.368, Effect size = 0.091) and (*P*-value = 0.135, Effect size = 0.182), respectively. For distal papilla, in SSA group, scores were constant through all time periods, so no statistical comparison was performed while in SHA group, there was no statistically significant change in distal papilla scores through different time periods (*P*-value = 0.135, Effect size = 0.182) (Table 2).

**Table 2:** Descriptive statistics and results of Mann–Whitney U test for comparison between mesial and distal papillae scores in the two groups and Friedman’s test for the changes within each group

**Variable**	**Time**	**SSA (*n*** **= 11)**		**SHA (***n* **= 11)**			***P*-value**	***Effect size (d)***
		Median (Range)	Mean (SD)	Median (Range)		Mean (SD)		
**Mesial papilla**	Base line	2 (1, 2)	1.91 (0.3)	1 (0, 2)		0.91 (0.7)	0.001*	1.653
	6 months	2 (2, 2)	2 (0)	1 (0, 2)		1.09 (0.54)	< 0.001*	1.922
	12 months	2 (2, 2)	2 (0)	1 (0, 2)		1.09 (0.54)	< 0.001*	1.922
	*P*-value	0.368		0.135				
	*Effect size (w)*	0.091		0.182				
**Distal papilla**	Base line	2 (2, 2)	2 (0)	1 (0, 2)	1 (0.77)		0.001*	1.564
	6 months	2 (2, 2)	2 (0)	1 (0, 2)	1.18 (0.6)		0.001*	1.564
	12 months	2 (2, 2)	2 (0)	1 (0, 2)	1.18 (0.6)		0.001*	1.564
	*P*-value	Not computed because the variable is constant		0.135				
	*Effect size (w)*			0.182				

^*^Significant at *P* ≤ 0.05

### Level and curvature of facial mucosa

At base line, after six as well as 12 months, there was no statistically significant difference in level and curvature of facial mucosa between the two groups (*P*-value = 0.061, Effect size = 0.647) and (*P*-value = 0.147, Effect size = 0.312) for each observation period, respectively. In both groups, scores were constant through all time periods, so no statistical comparison was performed (Table 3).

**Table 3:** Descriptive statistics and results of Mann–Whitney U test for comparison between level and curvature of the facial mucosa and root convexity/soft tissue color and texture scores in the two groups and Friedman’s test for the changes within each group

**Variable**	**Time**	**SSA (n = 11)**		**SHA (*n *= 11)**			***P*-value**	***Effect size (d)***
		Median (Range)	Mean (SD)	Median (Range)		Mean (SD)		
**Level of facial mucosa**	Base line	2 (1, 2)	1.91 (0.3)	2 (1, 2)		1.55 (0.52)	0.061	0.647
	6 months	2 (1, 2)	1.91 (0.3)	2 (1, 2)		1.55 (0.52)	0.061	0.647
	12 months	2 (1, 2)	1.91 (0.3)	2 (1, 2)		1.55 (0.52)	0.061	0.647
	*P*-value	Not computed because the variable is constant		Not computed because the variable is constant				
	*Effect size (w)*							
**Curvature of facial mucosa**	Base line	2 (2, 2)	2 (0)	2 (1, 2)	1.82 (0.4)		0.147	0.312
	6 months	2 (2, 2)	2 (0)	2 (1, 2)	1.82 (0.4)		0.147	0.312
	12 months	2 (2, 2)	2 (0)	2 (1, 2)	1.82 (0.4)		0.147	0.312
	*P*-value	Not computed because the variable is constant		Not computed because the variable is constant				
	*Effect size (w)*							
**Root convexity/** **soft tissue color and texture**	Base line	2 (1, 2)	1.73 (0.47)	1 (1, 2)		1.18 (0.4)	0.012*	1.042
	6 months	2 (1, 2)	1.73 (0.47)	1 (1, 2)		1.18 (0.4)	0.012*	1.042
	12 months	2 (1, 2)	1.73 (0.47)	1 (1, 2)		1.18 (0.4)	0.012*	1.042
	*P*-value	Not computed because the variable is constant		Not computed because the variable is constant				
	*Effect size (w)*							
**Total modified PES score**	Base line	10 (9, 10)	9.55 (0.52)	7 (4, 9)		6.45 (1.29)	< 0.001*	2.782
	6 months	10 (9, 10)	9.64 (0.5)	7 (6, 9)		6.82 (0.87)	< 0.001*	2.855
	12 months	10 (9, 10)	9.64 (0.5)	7 (6, 9)		6.82 (0.87)	< 0.001*	2.855
	*P*-value	0.368		0.052				
	*Effect size (w)*	0.091		0.27				

^*^Significant at *P* ≤ 0.05

### Root convexity/soft tissue color and texture

At base line, after six as well as 12 months, SSA group showed statistically significant higher root convexity/soft tissue color and texture scores than SHA group (*P*-value = 0.012, Effect size = 1.042) for each observation period. In both groups, scores were constant through all time periods, so no statistical comparison was performed (Table 3). 

### Total modified PES score

At base line, after six as well as 12 months, SSA group showed statistically significant higher total modified PES scores than SHA group (*P*-value < 0.001, Effect size = 2.782), (*P*-value < 0.001, Effect size = 2.855) and (*P*-value < 0.001, Effect size = 2.855), respectively. In both groups, there was no statistically significant change in total modified PES scores through different time periods (*P*-value = 0.368, Effect size = 0.091) and (*P*-value = 0.052, Effect size = 0.27), respectively (Table 3).

## Patient related outcomes

### The shape and the color of the implant tooth

At base line (1 week after crown delivery) as well as after 12 months, there was no statistically significant difference between the two groups regarding the shape of the implant tooth (*P*-value = 1, Effect size = 0) and color of the implant tooth (*P*-value = 0.317, Effect size = 0.154) for each observation period where the median (range) and mean (SD) of the scores of the shape of the implant tooth were 10 (9, 10) and 9.91 (0.3) for both groups. While the median (range) and mean (SD) of the scores of the color of the implant tooth were 10 (10, 10) and 10 (0) for SSA group and 10 (9, 10) and 9.91 (0.3) for SHA group.

### Gum appearance around the implant tooth

At base line (1 week after crown delivery) as well as after 12 months, SSA group showed statistically significantly higher gum appearance score than SHA group (*P*-value < 0.001, Effect size = 2.855) and (*P*-value < 0.001, Effect size = 3.011), respectively. In both groups, there was no statistically significant change in gum appearance scores after 12 months (*P*-value = 0.157, Effect size = 0.943) and (*P*-value = 0.564, Effect size = 0.353), respectively.

### Chewing ability on the implant tooth

At base line (1 week after crown delivery) as well as after 12 months, there was no statistically significant difference between the two groups (*P*-value = 0.403, Effect size = 0.312) for each observation period, respectively.

### Food impaction and cleansing efficiency around the implant tooth

At base line (1 week after crown delivery) as well as after 12 months, SSA group showed statistically significantly higher satisfaction with cleansing efficiency score than SHA group (*P*-value = 0.006, Effect size = 1.257) for each observation period, respectively.

### Overall satisfaction scores

At baseline (1 week after crown delivery) as well as after 12 months, SSA group showed statistically significantly higher overall satisfaction score than SHA group (*P*-value < 0.001, Effect size = 2.361) for each observation period, respectively.

Results of patient related outcomes are presented in Table 4.

**Table 4:** Descriptive statistics and results of Mann–Whitney U test for comparison between gum appearance, chewing and cleansing efficiencies, and overall satisfaction scores in the two groups and Wilcoxon signed-rank test for the changes within each group

**Variable**	**Time**	**SSA (*n *= 11)**		**SHA (*n *= 11)**			***P*-value**	***Effect ***
		Median (Range)	Mean (SD)	Median (Range)		Mean (SD)		***size (d)***
**Gum appearance**	Base line	10 (9, 10)	9.64 (0.5)	7 (6, 9)		7 (1)	< 0.001*	2.855
	12 months	10 (9, 10)	9.82 (0.4)	7 (6, 9)		7.09 (1.14)	< 0.001*	3.011
	*P*-value	0.157		0.564				
	*Effect size (w)*	0.943		0.353				
**Chewing ability**	Base line	9 (9, 10)	9.36 (0.5)	10 (9, 10)	9.55 (0.52)		0.403	0.312
	12 months	9 (9, 10)	9.36 (0.5)	10 (9, 10)	9.55 (0.52)		0.403	0.312
	*P*-value	Not computed because the variable is constant		Not computed because the variable is constant				
	*Effect size (w)*							
**Food impaction and cleansing efficiency**	Base line	10 (9, 10)	9.82 (0.4)	9 (7, 10)		8.64 (1.12)	0.006*	1.257
	12 months	10 (9, 10)	9.82 (0.4)	9 (7, 10)		8.64 (1.12)	0.006*	1.257
	*P*-value	Not computed because the variable is constant		Not computed because the variable is constant				
	*Effect size (w)*							
**Overall satisfaction**	Base line	9.8 (9.4, 10)	9.75(0.18)	9.2 (9, 9.6)		9.25 (0.22)	< 0.001*	2.361
	12 months	9.8 (9.2, 10)	9.8 (0.22)	9.2 (9, 9.6)		9.27 (0.24)	< 0.001*	2.361
	*P*-value	0.257		0.564				
	*Effect size (w)*	0.728		0.353				

^*^: Significant at *P* ≤ 0.05

## Discussion

Based on the previous findings, the null hypothesis is totally rejected as there were statistically significant differences between both tested groups regarding the peri-implant soft tissue profile and the patient related outcomes.

The customized socket sealing abutments were suggested to overcome limitations of the premanufactured standard healing abutments by transferring the natural tooth emergence profile to the final implant restorations [9, 10]. After one year of clinical evaluation period, no failures were discovered, and all patients attended all the clinical recall visits at different evaluation periods.

## Peri-implant soft tissue profile

The difference in the dimensions and the emergence profile design between SSA and SHA could mainly be the main reason behind the results of the present study which favored the SSA group in the peri-implant soft tissue profile. Ti SHA resulted in circular soft tissue contour throughout the healing process on the contrary to the SSA which posed larger gradually developing transmucosal area. This aimed to protect the bone grafting material from fast resorption rates until their full development and maturation, achieving highly aesthetic and biologically contoured implant restorations mimicking natural healthy teeth. Both the bone graft and the prosthetic socket seal together can augment the soft and hard tissue volume [17, 18].

The findings of modified PES score in the present trial are similar to other previous studies which followed comparable protocol to our study (SSA and bone graft socket filling) and showed significantly high PES scores compared to the standard healing abutments scores supporting the impact of the socket sealing abutment in preserving the peri-implant soft tissue enhancing the bio-esthetic outcomes [18, 20, 21]. While Perez et al., reported slight increase in the PES score of the customized healing abutment in comparison to that of the standard healing abutments after one year of the clinical follow up but with no statistically significant difference [13].

Throughout the different testing periods of the study, the distal and mesial papillae showed statistically significant higher scores in SSA group than SHA group. These findings may be attributed to the accurate soft tissue customization by the CAD-CAM PEEK SSA and the slightly concave subcritical contour of the SSA that offered more volume for the supra-crestal connective tissue and avoided the pressure on the interproximal soft tissues resulting in complete development and maturation of the interproximal papillae [15, 16].

It is essential to preserve the integrity of the interproximal papilla and minimize its loss throughout implant therapy. Maintaining the entire presence of the papilla helps prevent cosmetic issues (such as black triangles), problems with phonetics caused by air flow, and interproximal food accumulation [22]. However, in the posterior zone, it has only received intermittent attention. The presence of black triangles in the posterior region may not be worrying aesthetically, but it might contribute to food impaction, hence elevating the risk of peri-implantitis [23].

The findings of the papillae in our study came in agreement with a study that found out significantly increased papilla height in the customized healing abutment group than in the prefabricated group after one year of follow up [13].

Wang et al., proposed the use of anatomical healing abutments as an attractive option in implant prosthodontics. They found that these abutments efficiently raised the height of the papilla and reduced the occurrence of black triangles and peri-implantitis when employed in implant sites in the posterior region [24].

While Fernandes et al., reported no statistically significant difference in the papillae variation when the authors compared the customized healing abutment with collagen matrix sutured in situ after one year of clinical treatment [25].

In SHA group, there was slight increase in the mesial and distal papillae scores from baseline to 6 months follow up with no statistically significant difference. This improvement in papilla volume and height over long follow up periods has been investigated in some studies explaining the increase in the vertical height of the papilla over time in our study [26, 27].

Regarding the level and curvature of the facial mucosa, SSA group showed slightly higher scores than the SHA group with no statistically significant difference between both groups. The insertion of the collagen sponge plug to the post extraction sockets in cases of SHA group can be considered the reason behind the minimum changes in the vertical height of the gingival margin and the contour of the buccal mucosa in these cases [28]. Other influencing factors are the flapless minimally invasive tooth extraction, filling the gap between the implant and the bony socket walls with xenograft bone substitute, and ≥ 3 mm preoperative attached keratinized buccal mucosa [29].

The median (range) for the scores of the level of the facial mucosa was 2 (1, 2), so that the minimum score for both groups was 1 which means that the discrepancy in the gingival height was ≤ 1 mm that was considered as a minimal and insignificant change in the posterior unesthetic areas of the jaw as demonstrated in numerous studies [10, 30, 31].

The results of the level of the facial mucosa are consistent with Hu et al., that revealed no statistically significant difference with less midfacial mucosal recession in the customized group when they compared the customized healing collars with the traditional one in the premolar/molar post extraction sites [32]. While Fernandes et al., reported less alteration of the midfacial mucosal level and contour after one month then no statistically significant change after one year of clinical treatment when they evaluated the socket sealing abutments versus collagen plugs as a socket sealing technique for the maxillary immediate implants. Their study supports the comparable scores in the level and architecture of the buccal mucosa between our study’s experimental groups that showed the effect of using the collagen sponge plug with the Ti SHA in promoting the healing and re-epithelization of the surrounding implant tissues thus, raising their scores in the peri-implant mucosa [25].

SSA group showed statistically significantly higher root convexity/soft tissue color and texture scores than SHA group. These outcomes ensure the role of the customized anatomical socket sealing abutments in preserving the pre-extraction alveolar width with minimum discrepancies. Packing a bone xenograft into the space between the implant and the socket walls to bridge the gap, minimal traumatic tooth extraction and ≥ 1 mm preoperative buccal bone thickness might also play a role in these findings [29].

The results of root convexity/soft tissue color and texture came in agreement with Fabris et al., who showed substantial high alveolar ridge width in the cases of PEEK customized socket sealing abutment group after immediate implants in the maxillary premolar sites. Although the authors compared the customized anatomical abutments to submerged implants with cover screw and the jumping gap left empty without any bone grafting material [9].

Also, our results are aligned with a systematic review conducted by Lenz et al., that demonstrated considerable decrease in the alveolar process loss in cases of the anatomical healing abutments group relative to the prefabricated healing group [33].

However, our findings were opposed to those of other trial that did not detect any statistically significant change in the buccal alveolar bone thickness at the implant crestal level when the authors compared the conventionally constructed resin composite socket sealing abutment to the Ti prefabricated healing abutments [32]. This variation can be justified by including smokers and periodontal diseases in their study, the difference in the material of the socket sealing abutment which was conventionally fabricated resin composite that might cause irritation to the peri-implant tissues affecting the proper healing of the surrounding hard tissues. This in turn might hinder bone formation unlike the highly polished milled PEEK material applied in the current research [17, 34].

## Patient satisfaction

Many of the clinical studies assessing the impact of the immediate customized peri-implant profile on the esthetic and functional outcomes of the final delivered implant restorations had limited data concerning the patient related outcomes [30, 35]. Therefore, the present study aimed to examine the long-term effectiveness of the implant restorations regrading several patient related outcomes such as functional concerns (chewing and cleansing efficiency) and esthetic concerns (shape, color and gum appearance of the final restorations).

Concerning the gum appearance scores, patients showed statistically significantly higher satisfaction score in the SSA group than the SHA group after one year of the clinical follow up. These subjective outcomes came in support with the objective evaluation of higher modified PES scores for SSA group assuring the role of the customized healing abutments in promoting the peri-implant soft tissue esthetics.

While for the functional outcomes, the chewing efficiency scores showed no statistically significant difference between both groups unlike the cleansing efficiency which showed higher statistically significant satisfaction scores in the SSA group than in SHA group. Patients in the control group suffered from food impaction and difficulty in cleansing and removing the impacted food, this confirms that the customized profile of the SSA group resulted in a proper aligned contour of the final implant restorations with the bucco-palatal width of the alveolar ridge successively alleviated the symptoms of food impaction in the SSA group [21, 33].

The results of higher overall patient satisfaction scores in the SSA group than SHA group are comparable with those reported by Wang et al., who showed superior outcomes regarding patient satisfaction for the customized group compared to the conventional group but with no statistically significant change between the two groups [36].

## Limitations of the study

Short clinical observation period and the relatively small sample size may be considered a limitation as gathering data from larger group of patients over a longer clinical follow up period will enhance the reliability and validity of our results. Also, testing only one type of material in construction of the socket sealing abutment which was PEEK. More randomized controlled clinical trials with larger sample size and longer follow up period are recommended. Further studies are needed to evaluate the performance of different CAD-CAM materials of SSA against each other. Other trials can be conducted to compare the direct digital workflow in construction of the CAD-CAM SSA versus the indirect digital workflow which was followed in this trial.

## Conclusions

Within the limitations of the present study, the use of CAD-CAM PEEK SSA could drive the following conclusions:

1. Superior outcomes in terms of peri-implant soft tissue profile.
2. Enhanced patient satisfaction.

Therefore, CAD-CAM PEEK socket sealing abutments together with flapless minimally invasive tooth extraction and 3D guided implant placement can serve as a better alternative for Ti standard healing abutments.

## Clinical significance

PEEK SSA considered a viable option in reducing the implant prosthetic steps and procedures as it maintained the original emergence profile, eliminating the need for additional tissue conditioning. Dental practitioners should consider other factors in this treatment protocol rather than the contour of the SSA including the SSA material of construction, depth and 3D position of the implant, the vertical height of the mucosa, specific areas of implant insertion, implant diameter, implant abutment connection and patient compliance to the postoperative protocol.

## Data Availability

The data that support the findings of this study are available on request from the corresponding author.
